# Small fiber neuropathy and intractable scalp pruritus in dermatomyositis patients

**DOI:** 10.1002/ski2.173

**Published:** 2022-10-10

**Authors:** Pablo Vitoriano Cirino, Maria Hordinsky, Brian McAdams, Ricardo Romiti

**Affiliations:** ^1^ Department of Dermatology University of São Paulo Sao Paulo Brazil; ^2^ Department of Dermatology University of Minnesota Minneapolis Minnesota USA; ^3^ Department of Neurology University of Minnesota Minneapolis Minnesota USA

## Abstract

**Background:**

Scalp pruritus is a common symptom in Dermatomyositis (DM) patients. There are indications that small nerve fibers neuropathy could be involved in this symptom, however the etiology of scalp pruritus is not fully understood.

**Objectives:**

To assess epidermal nerve fiber (ENF) density of dermatomyositis patients with scalp pruritus by biopsy by confocal microscopy and immunohistochemistry with subsequent imaging analysis.

**Methods:**

DM patients with severe scalp pruritus from the dermatology outpatient clinic were compared to healthy volunteers. Two 4‐mm scalp skin biopsies were obtained above the right ear in the parietal region and below the occipital protuberance in the occipital region. Biopsy specimens were incubated with primary antibodies to protein gene product (PGP 9.5), calcitonin gene–related peptide (CGRP), substance P (SP) were used to visualize nerve fibers (ENF) and collagen IV was used to label the epidermal basement membrane. The number of ENFs per millimeter was counted and recorded as the mean of ± SD of counts in 16 images at two micrometer increments/sections, two from each of the samples. ENF densities were compared between groups and a multiple linear regression model was applied to associated factors with ENF density.

**Results:**

Fifteen DM patients with severe scalp pruritus and 12 healthy volunteers were included in the study. The mean number of ENF/mm in occipital region of DM group was 16.0 ± 13.9 while the control group in the same region was 99.8 ± 33.1. In parietal region the number of ENF/mm of DM group was 18.0 ± 20.7 while in control group was 50.4 ± 17.4 (*p* < 0.001).

**Conclusion:**

DM patients with pruritus could have some impairment of small nerve fiber density that could explain their recalcitrant scalp pruritus.

## Graphical abstract

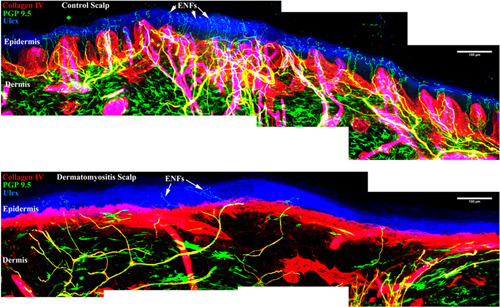

This is the first comparative study of epidermal nerve fibers analysis of dermatomyositis patients with scalp pruritus and healthy volunteers. Dermatomyositis patients had an important decrease in epidermal nerve fibers compared to healthy volunteers. The decrease of epidermal nerve fibers was associated with the DM group in both regions of the scalp but was also associated with being female and younger when we had analyzed the fronto‐parietal region.

1

**What is already known about this topic?**

- Dermatomyositis is an inflammatory condition that may be associated with recalcitrant treatment‐resistant pruritus.

**What does this study add?**

- Dermatomyositis patients showed reduced epidermal nerve fiber density compared with healthy controls.

**What is the translational message?**

- Small nerve fiber neuropathy could be an explanation for the recalcitrant scalp pruritus in Dermatomyositis patients.

## 2 INTRODUCTION

Dermatomyositis is a chronic autoimmune disease affecting mainly the skin and muscle but could affect other structures as blood vessels, joints, oesophagus, and the lungs.^1^, ^2^ The most prevalent cutaneous lesions include Gottron's sign, Gottron's papule, Shawl sign and heliotrope erythema.^2^, ^3^

Among symptoms involved in DM, scalp involvement is a common manifestation that includes diffuse, scaly erythema, atrophy, and often nonscarring alopecia.^4^, ^5^ Despite all the other signs and symptoms in the scalp, pruritus is one important cutaneous feature in DM and could help physicians to distinguish other diseases such as lupus erythematosus. Studies have shown prevalence of moderate to severe cutaneous itch between 38% and 50.8%^6^, ^7^ of DM cases.

Recently, some authors have reported cases of severe pruritus in the scalp of up to 71% of the patients with DM.^4^, ^5^, ^8^, ^9^ There are some indications that small nerve fibers of DM patients with scalp pruritus and to analyze associated factors to this symptom.

## 3 METHODS

It was an observational, collaborative study with patients diagnosed with DM and scalp pruritus recruited from Connective Tissue Disorders Unit of the University of São Paulo (Brazil) and compared with healthy volunteers recruited by the Department of Dermatology of University of Minnesota (USA). The study was approved by the Institutional Review Board Committee and written informed consent was signed by all participants.

Eligible criteria of DM patients with scalp pruritus were adults aged more than 18 years old that fulfilled diagnostic criteria for DM defined by Bohan & Peter^10^ modified by Drake et al.^3^ In addition, scalp pruritus without improvement by the existing treatments must be present. Participants with sensitive scalp, diabetes, frequent alcohol intake, previous chemotherapy treatment, drugs or diseases that could impact the peripheral nervous system were excluded to the study. The patient group was composed by afro‐descendants and Fitzpatrick 2–4 skin scale.

The healthy control group was composed also by afro‐descendants and Fitzpatrick 2–4 skin scale of the database of healthy patients without pre‐existing dermatology conditions of the outpatient clinic of the Dermatology Department of University of Minnesota that was previously submitted to the same method of analysis of the scalp.

### 3.1 Scalp biopsy method

After determining the biopsy sites, trichotomy and asepsis were done, followed by local anesthesia. Fresh scalp tissue was obtained by a 4 mm punch biopsy in the parietal region, two centimeters above the right ear and in the occipital region, above the occipital protuberance, in both groups.

### 3.2 Immunohistochemistry

The biopsy specimens collected in DM group were immediately fixed in cold Zamboni (paraformaldehyde–picric acid solution) for 24 h, then cryoprotected in 20% sucrose at pH 7.4 in phosphate‐buffered saline, packed in compliance with IATA DGR specifications with DRSm controlled temperature (4–8°C) package and shipped to the Department of Dermatology of University of Minnesota in Minneapolis where tissue sectioning and immunostaining were conducted.

All specimens were cut at 60 μm thickness, perpendicular to the surface of the skin with a freezing, sliding microtome and were incubated in buffer containing Triton X‐100 and normal donkey serum to reduce non‐specific background staining. Vertical sections of the biopsy specimens were incubated with primary antibodies to PGP 9.5 was used to visualise ENFs; and CGRP, and SP were used to visualize dermal nerve fibers and collagen IV labeled the epidermal junction membrane. The vertical sections were washed and incubated with fluorophore‐conjugated secondary antibodies (Jackson ImmunoResearch, Inc.). Species‐specific secondary antibodies conjugated to fluorophores Cy2 and Cy3 were used to label the primary antibodies.

### 3.3 Image analysis and confocal microscopy

Image stacks compromised of 16 images (z‐series) were acquired at 2 µm thin with a 20x objective lens using a spinning disk confocal microscope (Olympus BH‐2) employing Neurolucida® (MicroBrightField, Inc.) software. Merged dual colour z stacks images were used to trace ENFs that crossed the basement membrane of epidermis. After the tracing was completed and the basement membrane length was measured, the density of ENFs/mm of basement membrane was extracted with Neurolucida Explorer software. ENF density was calculated according to The European Federation of Neurological Studies task force guideline.^11^

#### 3.3.1 Statistical analysis

Quantitative numbers were analysed using the Exact Fisher test, according to the assumption check. Quantitative variables were expressed in mean and standard deviation, minimum, maximum, median and interquartile interval (25%–75%) and the groups were compared using *t*‐test or Wilcoxon‐Mann‐Whitney according to the assumption check of normality and equality of variances. Multiple linear regressions were applied to analyze associated factors to ENF density. Gender, age, and groups were adjusted to the final model. Stata/SE 12.0 for Mac was used to test the data.

## 4 RESULTS

During the study, 15 patients with DM and scalp pruritus were eligible for inclusion in the study, and they were compared to the total sample of healthy volunteers. Participants in the DM group were older than the healthy control group (55,9 ± 12,6 vs. 31,6 ± 10,4; *p* < 0.001); however, there was no difference in gender between groups. There were 13 (86.7%) female patients in the DM group vs. 7 (58.3%) in the healthy control group (*p* = 0.220).

**FIGURE 1:**
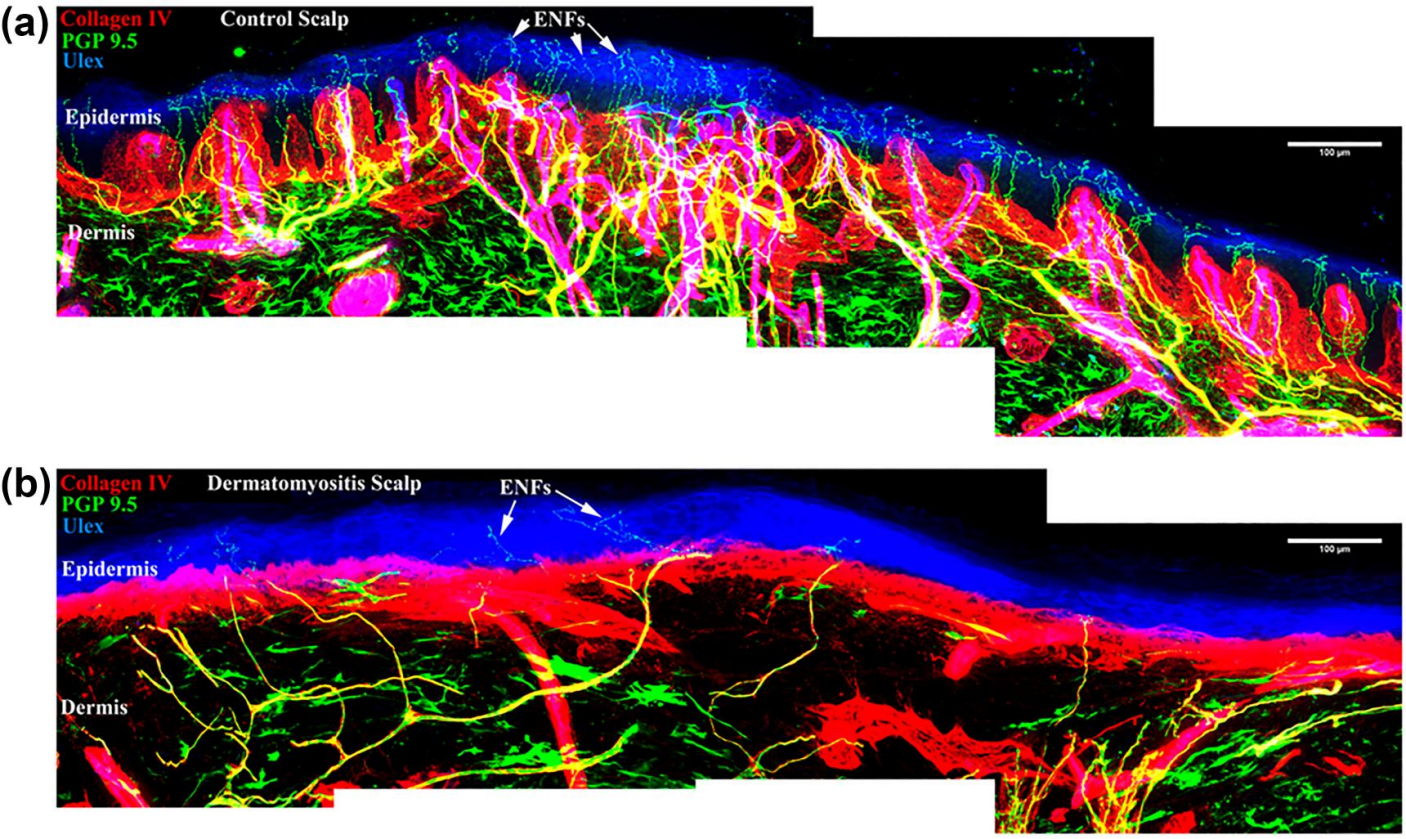
Confocal microscopy and immunohistochemistry of a healthy control patient (a) and Dermatomyositis (DM) patient (b). Presence of epidermal nerve fibers (ENFs) (arrows) in the (patient a). Collagen IV (basal membrane in red), PGP (pan‐neuronal marker in green), ULEX (epidermis in blue).

Abnormal nerve morphology and lower epidermal nerve density were observed DM skin (DM group) compared with normal skin (healthy control group), (Figure 1a,b) and confirmed by epidermal nerve fibers density in occipital region and parietal region (Table 1).

**TABLE 1:** Analysis of epidermal nerve fibre (ENF) density in fronto‐parietal and occipital regions in Dermatomyositis (DM) and healthy groups

	Occipital area			Fronto‐parietal area		
ENF	DM group (*n* = 15)	Healthy control group (*n* = 12)	*p* value	DM group (*n* = 14)	Healthy control group (*n* = 12)	*p* value
Mean	16.0 ± 13.9	99.8 ± 33.1		18.0 ± 20.7	50.4 ±1 7.4	
Median	14.8 (6.0–19.6)	99.5 (69.3–120.9)	<0.001	9.1 (6.4–12.1)	50.0 (35.2–64.9)	<0.001
Min;Max	0.9; 54.9	50.2; 162.4		1.5; 12.1	27.1; 77.5	

Abbreviations: DM, dermatomyositis; ENF, epidermal nerve fibers; IQ, interquartile interval; SD, standard deviation.

In a multiple linear regression, adjusted for the age, gender and group showed that ENF density in parietal was positively associated with increase of age and negatively to female and DM group. ENF density was 43.8 lower in the parietal region and 85.3 lower in occipital region of DM group when compared to control group. (*p* < 0.001) (Table 2).

**TABLE 2:** Multiple linear regression of epidermal nerve fibers (ENF) density in fronto‐parietal and occipital region

	Parietal		Occipital	
Variable	® adjusted (IC 95%)	*p* value	® adjusted (IC 95%)	*p* value
Age (year)	0.68 (0.01; 1.34)	0.048	0.18 (−0.77; 1.13)	0.695
Female (ref:men)	−18.2 (−36.0; −0.45)	0.045	−14.3 (−39.6; 10.9)	0.252
DM group (ref: control)	−43.8 (−64.9; −22.7)	<0.001	−85.3 (−115; −55.3)	<0.001

*Note*: Values adjusted to age, female and group.

## 5 DISCUSSION

The study of cutaneous scalp innervation was neglected for many years particularly with direct comparison of normal to pathological conditions affecting epidermal innervation. This lack of knowledge about the pathophysiology of scalp pruritus in DM patients and the absence of a detailed description of nerves and the peripheral nervous system in the scalp of DM subjects, led to the authors to endeavor to analyze the epidermal innervation in the scalp of DM patients and compare them to a healthy control group. Thereby, this is the first study that compared a group with DM and scalp pruritus to healthy controls. In our results, we have shown that the DM group had a significant reduction of epidermal nerve fiber density in occipital and parietal region (Table 1).

Pruritus is an extremely common and complex symptom among neuropathic diseases and has an important impact on the quality of life.^6^, ^12^ It is a skin irritation that causes an intense need to scratch the affected skin, bringing temporary and immediate relief. Its function is to protect against some external agents potentially harmful to the skin.^12^ Contrary to recent advances in the understanding of the physiopathology of pruritus, the bases of itching in the scalp of DM patients had not been clarified. The hair follicle is densely innervated and there is an abundant dermal vascularity with a unique innervation which, when some peripheral neuropathy occurs, the patient could be affected with dysesthesias and pruritus.^13^ In patients with DM, inflammatory myopathy also occurs in association with microvascular alteration; thus, it is possible that one or many of these structures of the scalp could be altered leading to altered sensation and causing itching. In our sample, all DM patients were symptomatic at the time of biopsy with signs of itching associated with abrasions, hematic crusts and in some of them even cicatricial alopecia due to intense trauma caused by scratching.

In our study, we chose the indirect immunofluorescence technique with confocal microscopy. Despite being a more complex method, it has the advantage of identifying multiple antigens in the same structures, permitting the axons and basement membrane visualization of antibodies against PGP 9.5 and collagen IV.^15^

Cutaneous nerve fibers in the scalp are organized into small nerve bundles, located just below the dermoepidermal junction, in the subepidermal plexus, which branches to form the epidermis.^16^ Small nerve fib2 out of 3 signs and/or symptoms are present: (i) decrease in tactile or thermal sensitivity and/or allodynia/hyperalgesia, (ii) abnormal heat and cold sensitivity test and/or (iii) decrease of ENF density.^17^ However the gold standard criteria is the reduction of epidermal nerve fiber density.^18^, ^19^ In the present study, all patients studied presented criteria for SNF neuropathy with an important and significant decrease in the ENF density.

In our study, the morphology was also quite altered, with tangles of thick nerves in the subepidermal neural plexus, axonal edema and few branches to the epidermis. Among the ENF, low density and little branching were found as shown in Figure 1.

The reduction of small nerve fibers density is also present in many other diseases such as lupus erythematosus,^20^ diabetes, Fabry’s disease, familial dysautonomia, HIV, post neurotoxic drugs use and congenital insensitivity of pain.^14^, ^15^ A recent cohort of 1827 systemic lupus erythematosus patients followed up in 31 clinics in Europe, Asia, and North America have shown that 7.6% patients presented different types of PNS disease. Among them, peripheral neuropathy was the predominant type of PNS disease (41.0%–66/161) and was associated with a significantly lower quality of life when compared to those patients without PNS disease.^21^ This reduction could be the result of selective small fibre neuropathies or as part of a process that involves both large^20^ and smaller fibres associated with an impairment of reflex mechanisms of vasoconstriction and vasodilation of peripheral blood vessels, as shown in previously studies.^17^

Prior to this study, there was only a case report that had shown the reduction of small nerve fibers density^9^ in DM patients, suggesting that a scalp neuropathy may be present in individuals with dermatomyositis and recalcitrant scalp pruritus. Based on this report, the present study was designed to have a better comprehension of ENF density in DM patients with scalp pruritus, something that our results have shown.

In multiple linear regression, after adjusted for possible confounders, we observed that being a DM patient, reduces the ENF density in 43.8 ENF/µm, in fronto‐parietal area and in 85.3 ENF/µm in occipital area. In the fronto‐parietal area, being a woman reduces in 18.2 ENF/µm. These data express the importance of the DM in the reduction of ENF density, regardless of other factors, mainly in the occipital area.

Other factors such as age and gender were associated with ENF density only in the fronto‐parietal area in this study. Two factors could explain these results. First, the innervation of the two areas are different. While the occipital area is innervated by greater occipital nerve, fronto‐parietal area is innervated by other nerves as auriculotemporal, zygomaticotemporal, supraorbital and supratrochlear nerve. Thus, the behavior of the pruritus could be different. Another possible cause could be the elevated inflammation of the scalp in older DM patients. During the biopsies, we have observed that older DM patients presented more inflammation in the fronto‐parietal than in the occipital area. Then, this difference in symptomatology between areas could also explain the statistical differences between the areas.

The fact we could not match the groups by gender and age and that we had a small sample could bias the results. However, in a post hoc analysis conducted after the final result showed a sample power of more than 99% with an error of 1% in ENF density difference between groups in both areas. Then, it is possible to affirm that we had robust results. Moreover, based on the complexity and the costs involved in the method, the number of samples analyzed was higher than the previous study.^9^

In DM and control groups we had afro‐descendants and Fitzpatrick skin types 2–4 showing that we had a similar racial miscegenation, with no significant difference in ENF density in control group. Regarding the possibility of genetic factor have any effect on nerve fiber density, there’s no information in current studies indicating that different genetic information could significantly alter the human skin innervation. The rarity of this disease as well as the scalp symptom intensity and the high costs to develop this research limited our study.

In conclusion, we demonstrated an important reduction in ENF density in occipital and fronto‐parietal areas in DM patients compared to healthy controls, suggesting that dermatomyositis could be associated with small fiber neuropathy. This data opens new paths to diagnosis, treatment and follow up of these and consequently, could improve their quality of life.

This was an international collaboration between Brazil and the United States that had involved dermatologists from the University of São Paulo and University of Minnesota (UMN) and a team of neurologists in the Kennedy Laboratory at the UMN. We acknowledge Prof. Dr. William Kennedy, a great and inspiring researcher who guided this study of skin’s innervation.

## CONFLICT OF INTEREST

The authors declare that there is no conflict of interest that could be perceived as prejudicing the impartiality of the research reported.

## AUTHOR CONTRIBUTIONS

**Pablo Vitoriano Cirino**: Conceptualization (Equal); Data curation (Equal); Formal analysis (Equal); Investigation (Equal); Methodology (Equal); Validation (Equal); Writing – original draft (Equal); Writing – original draft (Equal). **Maria Hordinsky**: Investigation (Equal); Methodology (Equal); Project administration (Equal). **Brian McAdams**: Data curation (Equal); Formal analysis (Equal); Methodology (Equal); Writing – original draft (Equal). **Ricardo Romiti**: Methodology (Equal); Project administration (Equal); Supervision (Equal); Validation (Equal); Writing – original draft (Equal).

## ETHICS STATEMENT

The research ethics committee and the institutional review board of the University of São Paulo‐Brazil, followed by the national health authorities approved the research by the approval number: 4633738.

## Data Availability

The data that support the findings of this study are available on request from the corresponding author. The data are not publicly available due to privacy or ethical restrictions.
